# IMPROVING ASSISTED LIVING RESIDENTS QUALITY OF LIFE: RESULTS FROM AN MN ASSISTED LIVING REPORT CARD

**DOI:** 10.1093/geroni/igae098.0502

**Published:** 2024-12-31

**Authors:** Tetyana Shippee, Mark Woodhouse, Tricia Skarphol, Kelly Moeller, Lauren Glass, Rachel Shands

**Affiliations:** University of Minnesota School Of Public Health, Minneapolis, Minnesota, United States; University of Minnesota, Minneapolis, Minnesota, United States; University of Minnesota, Minneapolis, Minnesota, United States; University of Minnesota, Minneapolis, Minnesota, United States; Minnesota Department of Human Services, St. Paul, Minnesota, United States; Minnesota Department of Human Services, St. Paul, Minnesota, United States

## Abstract

In 2019, Minnesota enacted new legislation resulting in creation of an online Assisted Living Report Card. As part of this work, two surveys, the Resident Quality of Life Survey and Family Satisfaction Survey, were developed to assess AL residents’ quality of life (QOL) and family satisfaction. Our study presents the development and analysis of these surveys in Minnesota AL communities from 2020-2023. Resident respondents (N=12,091) were predominantly female (68.6%) and White (81.4%), with mean age 82.8 years; family satisfaction survey respondents (N=11,935) were mostly female (64.2%) and White (91.1%), with a mean age 63 years. Respondents rated various survey items with scores from 0 (lowest) to 2 (highest) for resident QOL, and 0 (lowest) to 3 (highest) for family satisfaction, with mean scores calculated for each of the domains. Overall, resident quality of life and family satisfaction scores were high, yet “overall satisfaction” (1.22), “engagement” (1.48), and “food” (1.53) domains had the lowest scores, while “environment” (1.88), “finances” (1.85), and “security” (1.82), had the highest mean scores among residents. Among family members, having one’s “personal needs met” (2.12) and “finances” (2.13) had the lowest mean scores, while “choice” (2.36), “housekeeping” (2.38), and “environment” (2.39) had the highest mean scores. Insights from our study highlight areas for improvement to enhance well-being and satisfaction among AL residents and their families.

